# Commentary: Vitamin D Status in Relation to the Clinical Outcome of Hospitalized COVID-19 Patients

**DOI:** 10.3389/fmed.2022.922820

**Published:** 2022-06-16

**Authors:** Marijn M. Speeckaert, Joris R. Delanghe

**Affiliations:** ^1^Department of Nephrology, Ghent University Hospital, Ghent, Belgium; ^2^Research Foundation-Flanders (Fonds Wetenschappelijk Onderzoek), Brussels, Belgium; ^3^Department of Diagnostic Sciences, Ghent University, Ghent, Belgium

**Keywords:** COVID-19, vitamin D binding protein (DBP), mortality, vitamin D, polymorphisms

## Introduction

With interest, we read the paper of Hafez et al. (1), which investigated the relationship between vitamin D status and clinical outcome of hospitalized coronavirus disease 2019 (COVID-19) patients. An increased risk for intensive care unit (ICU) admission and in-hospital mortality were associated with (very) low serum 25-hydroxyvitamin D [25(OH)D] concentrations. However, we would like to discuss the potentially important influence of vitamin D binding protein (DBP) and its polymorphism on this observation.

## The Polymorphic Character of Vitamin D Binding Protein

DBP is the primary transporter and reservoir for the major vitamin D metabolites, which are mostly protein bound. Eighty-five to eighty-eight percent of circulating vitamin D is bound to DBP, 12–15% is bound to albumin, and the remaining fraction circulates in its free form. Humans have a significant DBP polymorphism with three well-known alleles [DBP1S (slow), DBP1F (fast), and DBP2], and more than 120 rare variants (2). The genetic polymorphisms rs7041 and rs4588 define the DBP alleles: DBP1S [rs7041G (ASP), rs4588C (Thr)], DBP1F [rs7041T (ASP), rs4588C (Thr)], and DBP2 [rs7041T (ASP), rs4588A (Lys)] (3). The median plasma concentration of 25(OH)D and 1,25-dihydroxyvitamin D [1,25(OH)_2_D] are determined by the DBP phenotypes. Patients with a DBP1-1 phenotype have higher vitamin D concentrations in comparison with DBP2-1 individuals, which in turn have a higher amount of vitamin D compounds compared to the DBP2-2 group. A 5-fold difference in the mean serum DBP concentration is present among the 3 common DBP phenotypes (DBP1-1 > DBP2-1 > DBP2-2) (4). In genome-wide association studies (GWAS), rs2282679, an intronic polymorphism in the *DBP* gene, has been linked to decreased 25(OH)D and DBP levels in several different populations. rs2282679 is a near-perfect proxy (i.e., substitute) for the coding single nucleotide polymorphism (SNP) rs4588. The C-allele of rs2282679 is typically coinherited with the A-allele of rs4588 and vice versa. Because the C-allele of rs2282679 is associated with lower 25(OH)D and DBP levels, rs2282679-C/C allele carriers have lower vitamin D and DBP levels than carriers of one rs2282679-C-allele, who have lower vitamin D and DBP levels than rs2282679-A/A patients (5). In addition, increasing copies of the minor allele of rs705117 in the *DBP* gene is associated with lower serum DBP concentrations (6).

## Vitamin D Binding Protein Phenotypes and COVID-19

The observed link between vitamin D deficiency and poor clinical outcomes among COVID-19 patients in the paper of Hafez et al. (1) could be influenced by DBP and its polymorphism. In a previous study, we looked at the impact of DBP phenotypes in patients with a severe acute respiratory syndrome coronavirus 2 (SARS-CoV-2) infection. In 55 countries, the DBP1 allele (a combination of DBP1F and DBP1S) frequency was shown to be inversely related to COVID-19 prevalence and mortality (7). The Metabolism score encoded by the *DBP* and the *CYP24A1* genes was associated with infection severity in a study of 517 Portuguese COVID-19 patients. A more in-depth examination revealed that the polymorphism DBP rs2282679 might account for the majority of the intriguing link found (8). So carriers of a DBP polymorphism corresponding with lower vitamin D and DBP concentrations might have a higher risk of an infaust prognosis due to a SARS-CoV-2 infection. In a recent study, median serum DBP concentrations among COVID-19 patients were significantly lower in non-survivors in comparison to survivors in univariate analysis (9).

## Discussion

Although the reported findings by Hafez et al. (1) could be partly explained by the protective immunomodulatory effects of vitamin D (7), also DBP might exert its influence on the outcome of COVID-19 patients. In addition to its unique sterol binding ability and its influence on the concentration of vitamin D metabolites, DBP performs a number of critical biological tasks such as actin scavenging, chemotaxis, macrophage activation, and fatty acid transport, which might explain these observations (4). The most prevalent manifestation of severe COVID-19 is viral pneumonia-induced acute respiratory distress syndrome (ARDS) (10). An abnormal increase in pulmonary vascular permeability is one of the characteristics of ARDS with loss of barrier integrity mediated by cell-cell contact breakdown and actin remodeling (11). Actin release, which is implicated in microvascular damage, is a feature of ARDS, as well as organ failure and septic shock. Besides monomeric globular (G-actin), extracellular polymerized filamentous actin (F-actin) is generated in conjunction with coagulation factor Va, causing disseminated intravascular coagulation and multiple organ failure syndrome. The intravascular actin scavenging system, which consists of gelsolin and DBP, cleaves actin and prevents repolymerization to counteract these procoagulant effects. Gelsolin severs and depolymerizes actin filaments, but DBP can impede new filament production and sequester actin due to its high affinity for G-actin. Several investigations have shown that the actin scavenging system plays a crucial role during severe sepsis and respiratory failure (12). Significantly lower plasma DBP levels have been measured in ARDS patients in comparison with healthy controls (13).

In conclusion, increased severity levels in COVID-19 individuals may be explained by a genetic predisposition to vitamin D deficiency caused by distinct DBP polymorphisms. Although it is unknown if these links are causal or consequential, they are potential candidates for further research.

## Author Contributions

MS wrote the first draft of the manuscript. JD edited and revised the manuscript for important content. All authors re-read, edited, and approved the final version of the manuscript for submission.

## Conflict of Interest

The authors declare that the research was conducted in the absence of any commercial or financial relationships that could be construed as a potential conflict of interest.

## Publisher's Note

All claims expressed in this article are solely those of the authors and do not necessarily represent those of their affiliated organizations, or those of the publisher, the editors and the reviewers. Any product that may be evaluated in this article, or claim that may be made by its manufacturer, is not guaranteed or endorsed by the publisher.
